# Catechin inhibits ox-LDL-induced ferroptosis in vascular smooth muscle cells to alleviate and stabilize atherosclerosis

**DOI:** 10.3389/fnut.2025.1594708

**Published:** 2025-06-02

**Authors:** Minghua Guo, Lingli Xie, Huanhuan Yuan, Duan-fang Liao, Xi-Long Zheng

**Affiliations:** ^1^School of Integrated Chinese and Western Medicine, Hunan University of Chinese Medicine, Changsha, Hunan, China; ^2^Key Laboratory of Hunan Province for Integrated Traditional Chinese and Western Medicine on Prevention and Treatment of Cardio-Cerebral Diseases, School of Integrated Chinese and Western Medicine, Hunan University of Chinese Medicine, Changsha, Hunan, China; ^3^Division of Stem Cell Regulation and Application, School of Pharmacy, Hunan University of Chinese Medicine, Changsha, Hunan, China; ^4^Department of Biochemistry and Molecular Biology, Libin Cardiovascular Institute, Cumming School of Medicine, University of Calgary, Calgary, AB, Canada

**Keywords:** catechin, ferroptosis, vascular smooth muscle cells, oxidative stress, Nrf2 pathway, GPX4, atherosclerosis, ox-LDL

## Abstract

Atherosclerosis (AS) is a chronic, progressive vascular disease marked by lipid deposition in the arterial intima, vascular wall thickening, luminal narrowing, and compromised blood flow. Although macrophage-derived foam cells are well-studied, vascular smooth muscle cells (VSMCs) also substantially contribute to AS, particularly when they transition into foam cells under oxidative stress. Accumulating evidence suggests that ferroptosis—an iron-dependent, regulated cell death mechanism characterized by lipid peroxidation—exacerbates AS pathology through oxidative damage and vascular dysfunction. Catechin, a potent antioxidant abundant in green tea, has demonstrated efficacy in reducing oxidative stress; however, its role in suppressing VSMC ferroptosis induced by oxidized low-density lipoprotein (ox-LDL) remains unclear. Here, we evaluated catechin's capacity to protect VSMCs against ox-LDL-induced ferroptosis, focusing on its modulation of the Nrf2/SLC7A11/GPX4 axis. Mouse vascular smooth muscle (MOVAS) cells were incubated with ox-LDL to induce foam cell formation and ferroptosis. We assessed intracellular iron, lipid peroxidation, reactive oxygen species (ROS), and antioxidant defenses and examined mitochondrial ultrastructure via transmission electron microscopy (TEM). Ferroptosis-related proteins were measured by Western blot, immunofluorescence, and qPCR. *In vivo*, ApoE^−/−^ mice on a high-fat diet (HFD) underwent partial carotid ligation with local catechin administration to investigate plaque formation and ferroptosis in arterial tissue. Our results show that catechin reduced intracellular Fe^2+^, decreased ROS and malondialdehyde (MDA) levels, and preserved mitochondrial integrity in ox-LDL-exposed MOVAS cells. Catechin also enhanced GSH and SOD levels and restored GPX4, SLC7A11, and Nrf2 expression, thereby reducing foam cell formation. In ApoE–/– mice, catechin reduced plaque size, mitigated lipid deposition, and upregulated GPX4, SLC7A11, and Nrf2 in the arterial wall. Collectively, these findings confirm that catechin prevents ox-LDL-induced ferroptosis in VSMCs by activating the Nrf2/SLC7A11/GPX4 pathway, highlighting its potential therapeutic value for atherosclerosis. This study provides additional evidence for the role of dietary polyphenols in regulating ferroptosis within VSMCs.

## 1 Introduction

Atherosclerosis (AS) remains a principal driver of cardiovascular disease (CVD) (1). Pathologically, it involves chronic endothelial damage, inflammatory cell infiltration, and lipid-laden plaque formation within arterial walls (2, 3). While macrophage-derived foam cells have been intensively studied, mounting evidence shows that VSMC-derived foam cells also play a critical role in plaque expansion and destabilization (4). Under inflammatory conditions and exposure to ox-LDL, VSMCs may accumulate lipids, form foam cells, and contribute to fibrous cap formation as well as vulnerability (4–7).

Ferroptosis, discovered relatively recently, is an iron-dependent mode of regulated cell death typified by lipid peroxidation and associated with excess reactive oxygen species (ROS) overload (2, 8). Studies indicate that ferroptosis aggravates plaque progression by promoting foam cell formation and local oxidative stress (9–12). The antioxidant enzyme glutathione peroxidase 4 (GPX4) detoxifies lipid peroxides and is a key negative regulator of ferroptosis (13). SLC7A11 (also called xCT) prevents ferroptosis by facilitating cystine import, which supports glutathione (GSH) synthesis (14). Nrf2, a master regulator of cellular redox homeostasis, can transcriptionally activate GPX4, SLC7A11, and other antioxidant genes, thus restricting ferroptosis (15–17).

Catechin, a polyphenolic compound abundantly present in green tea, exerts strong antioxidant effects by scavenging ROS, enhancing endogenous antioxidant enzymes, and improving lipid metabolism (18, 19). Although its inhibitory effect on VSMC ferroptosis under ox-LDL challenge is poorly characterized, we hypothesized that catechin would reduce ferroptosis through the Nrf2/SLC7A11/GPX4 axis, thereby diminishing lipid peroxidation and stabilizing atherosclerotic lesions. Using MOVAS cells and a partially ligated ApoE^−/−^ mouse model, we investigated catechin's ability to inhibit ferroptosis and foam cell formation, measuring intracellular iron, lipid peroxidation, antioxidant defenses, mitochondrial integrity, and ferroptosis-related proteins. Our findings provide novel insights into catechin's anti-ferroptotic and plaque-stabilizing effects (20).

## 2 Materials and methods

### 2.1 Chemicals and reagents

Catechin (Solarbio Science and Technology Co., Ltd., Beijing, China) and ox-LDL (23 nmol MDA/mg protein; Yiyuan Biotechnologies, Guangzhou, China) were used. Dulbecco's modified Eagle's medium (DMEM), phosphate-buffered saline (PBS), and fetal bovine serum (FBS) were obtained from routine suppliers. Cell Counting Kit-8 (CCK-8) was purchased from Dongren Chemical Technology Co., Ltd. (Dongying, China). Colorimetric assay kits for superoxide dismutase (SOD), malondialdehyde (MDA), glutathione (GSH), total cholesterol (TC), and free cholesterol (FC) were from Jiancheng Biotechnology (Nanjing, China). FerroOrange (Dojindo, Japan) was used to detect intracellular Fe^2+^. Primary antibodies against Nrf2, GPX4, SLC7A11, and GAPDH were from ABclonal Biotechnology Co., Ltd. (Boston, MA, USA).

### 2.2 Cell culture

Mouse aortic vascular smooth muscle cells (MOVAS, ATCC, CRL-2797) were obtained from the American Type Culture Collection and cultured in DMEM supplemented with 10% FBS at 37 °C in a 5% CO_2_ environment. Cells were subcultured when reaching ~80% confluence and used within 10 passages (21, 22).

### 2.3 Animal model

Twenty-eight-week-old ApoE–/– male mice were purchased from Nanjing Junke Bioengineering Co., Ltd. [Nanjing, China; license no. SCXK (Su) 2022–0001]. All animal experiments were approved by the Ethics Committee of Hunan University of Chinese Medicine (Approval No. SLBH-202401290001). They were used for the partial carotid ligation model, in which catechin was administered via local application using hydrogel. Mice were randomly divided into two groups (*n* = 10 per group): a mock-treatment group (Mod), treated locally with a hydrogel containing normal saline, and a catechin-treated group (CAT), receiving local application of a hydrogel containing catechin at a concentration of 1 mM. Partial ligation of the left carotid artery (LCA) was performed by ligating the left external carotid artery (ECA), left internal carotid artery (ICA) and occipital artery (OA) using 9–0 Ethalin sutures while leaving the superior thyroid artery (STA) intact. Immediately after ligation, the respective hydrogel formulation was applied locally around the perivascular region, followed by wound closure. Postoperatively, mice were placed on a high-fat diet (21% milk fat and 1.25% cholesterol) for 4 weeks before further analyses. Ultrasound was used to confirm successful carotid ligation by measuring changes in blood flow in the LCA.

### 2.4 Cell viability assay

MOVAS cells (5 × 103 cells/well in 96-well plates) were incubated with catechin (0, 25, 50, 100, 200, 400 μM) for 12–48 h. Cell viability was measured using the CCK-8 assay at 450 nm.

### 2.5 Foam cell formation and lipid content

MOVAS cells (1 × 10^5^ cells/well in 12-well plates) were serum-starved for 24 h, then pretreated with catechin (100 or 200 μM) for 24 h before exposure to ox-LDL (0~100 μg/mL) for another 24 h (23). Total cholesterol (TC) and free cholesterol (FC) were measured by colorimetric kits, and esterified cholesterol (CE) was calculated as the difference between TC and FC. Oil Red O staining was performed on cells fixed in 4% paraformaldehyde (24).

### 2.6 FerroOrange staining for intracellular iron

MOVAS cells (1 × 10^6^ cells/well in 6-well plates) were treated with catechin plus ox-LDL. After 24 h, they were incubated in serum-free, phenol red-free DMEM containing 1 μM FerroOrange for 30 min at 37 °C. Fluorescence images were acquired and quantified with ImageJ (25).

### 2.7 ROS and oxidative stress markers

Intracellular ROS was measured by flow cytometry using the fluorescent probe DCFH-DA (10 μM, incubated for 20 min at 37°C). After incubation with DCFH-DA, MOVAS cells were washed three times with serum-free medium. Unstained cells served as negative controls for gating. We used MDA levels as an index of lipid peroxidation. SOD and GSH levels were determined to evaluate antioxidant capacity (26, 27).

### 2.8 Transmission electron microscopy (TEM)

MOVAS cells (1 × 10^6^ cells/well) were fixed in 2.5% glutaraldehyde for > 24 h, post-fixed in 1% osmium tetroxide, and dehydrated in graded ethanol. Ultrathin sections were stained with 2% uranyl acetate and lead citrate. Mitochondrial ultrastructure was observed with a Hitachi H-7500 TEM (28).

### 2.9 Western blot analysis and immunofluorescence

Total proteins were lysed in RIPA buffer supplemented with protease inhibitors. Protein concentration was quantified using a BCA assay. Equal protein amounts (20–40 μg) were separated by 12% SDS-PAGE and transferred to PVDF membranes. Membranes were blocked, incubated with primary antibodies overnight (1:1,000), then secondary HRP-conjugated IgG (1:5,000). Protein bands were visualized by chemiluminescence and quantified using ImageJ software.

For immunofluorescence, arterial sections (6–8 μm) were permeabilized with 0.3% Triton X-100, and blocked with 5% BSA. Primary antibodies against GPX4 (1:200), SLC7A11 (1:200), Nrf2 (1:200), and α-SMA (1:200) were applied overnight at 4°C, and then incubated with fluorophore-conjugated secondary antibodies. Nuclei were stained with DAPI, and images were taken by confocal microscopy.

### 2.10 *In vivo* tissue collection and histology

Two weeks after partial carotid ligation, mice were euthanized by CO_2_ asphyxiation. Carotid arteries and aortic tissues were dissected, fixed in 4% paraformaldehyde, and stained en face with Oil Red O or embedded in OCT for cryosectioning. Sections were stained with Oil Red O or H&E for histological assessment. Plaque area was quantified by ImageJ (29).

### 2.11 Lipid profile

Serum levels of total cholesterol, triglycerides, HDL-c, and LDL-c were measured with colorimetric kits as described above, and absorbances were recorded.

### 2.12 Statistical analysis

ImagePro Plus 6.0 and GraphPad Prism 5.0 software were used for image analysis and data visualization, respectively. Statistical analysis was performed using SPSS 22.0 software. Data are presented as mean ± standard deviation ($\bar{\text{x}}$ ± s). Differences among multiple groups were analyzed using one-way ANOVA, while differences between two groups were analyzed using the *t*-test. The Shapiro–Wilk test was used to assess the normality of data prior to applying *t*-tests or ANOVA. A *P*-value < 0.05 was considered statistically significant.

## 3 Results

### 3.1 Titration of catechin concentrations and optimal ox-LDL dose for foam cell formation

**Figure 1:**
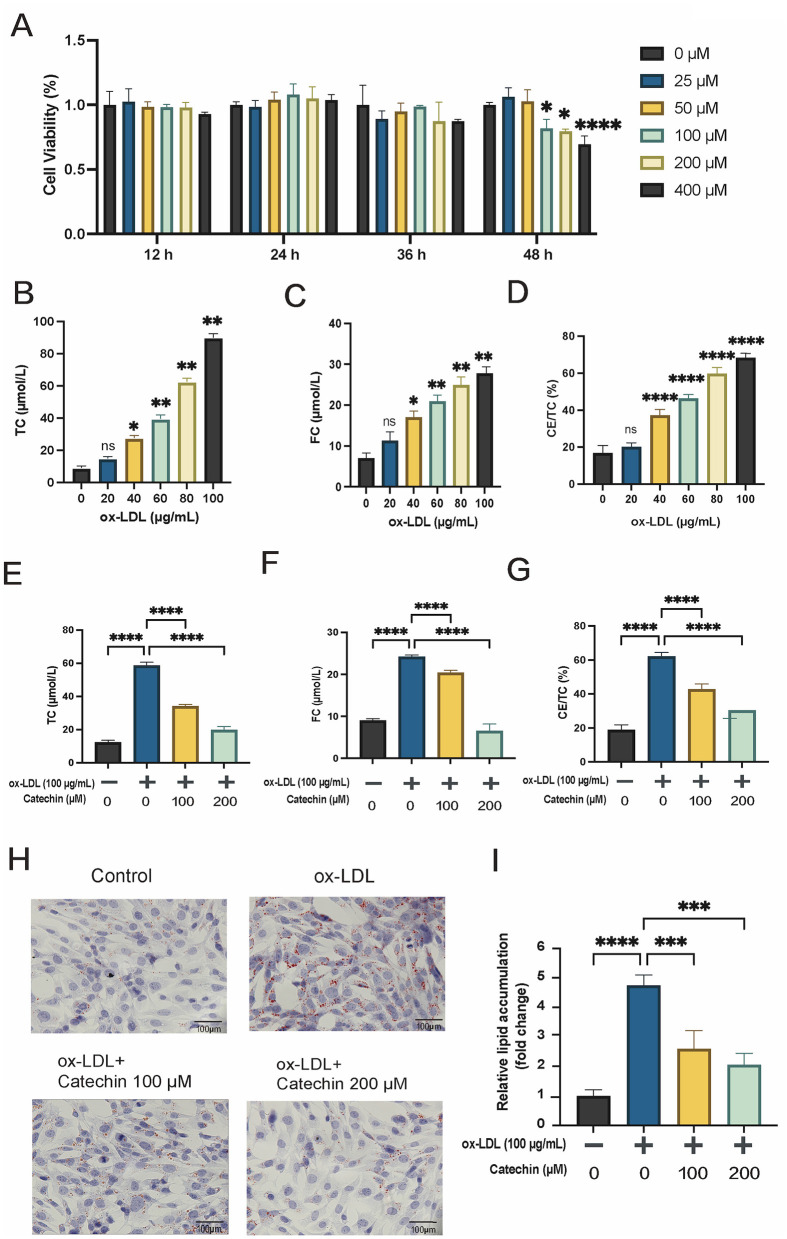
Catechin's effect on MOVAS viability, optimal ox-LDL dose, and foam cell formation. **(A)** A CCK-8 assay shows that catechin at concentrations >200 μM significantly reduces MOVAS viability at 36–48 h. **(B–D)** Increasing ox-LDL doses (0–100 μg/mL) increase total cholesterol (TC), free cholesterol (FC) levels, as well as the cholesteryl ester to total cholesterol (CE/TC) ratio. At ox-LDL concentrations ≥80 μg/mL, ox-LDL induces an esterification rate (CE/TC), and the CE/TC ratio exceeds 50%, indicating foam cell formation. **(E–G)** Co-incubation with catechin (100 or 200 μM) lowers total and free cholesterol levels, reducing the esterification CE/TC ratio to below 50%. **(H, I)** Oil Red O staining shows the extensive lipid droplets in the ox-LDL group, and this accumulation is reduced by catechin treatment. Data are mean ± SD (*n* = 5). ns, not significant; *p < 0.05, **p < 0.01, ***p < 0.001, ****p < 0.0001.

We first determined the appropriate concentration range of catechin for MOVAS cells. Lower catechin concentrations (25–100 μM) did not significantly affect cell viability for up to 48 h, whereas higher concentrations (200 and 400 μM) reduced viability at 36–48 h (*p* < 0.01; Figure 1A). Based on these findings, 100–200 μM was selected for subsequent 24-h treatments.

To induce foam cell formation, we tested ox-LDL at 0, 20, 40, 60, 80, and 100 μg/mL in MOVAS for 24 h. An esterification ratio (CE/TC) exceeding 50% indicated successful foam cell formation. When ox-LDL reached 80 μg/mL, the CE/TC ratio surpassed 50%, and 100 μg/mL produced a robust foam cell phenotype (Figures 1B–D). Consequently, 100 μg/mL was employed in all subsequent experiments.

### 3.2 Catechin decreases lipid accumulation and foam cell formation

We next examined whether catechin attenuates ox-LDL-induced lipid accumulation. MOVAS cells were incubated with 100 μg/mL ox-LDL, with or without catechin (100 or 200 μM), for 24 h. Catechin significantly lowered total cholesterol, free cholesterol, and the CE/TC ratio relative to ox-LDL alone (*p* < 0.01; Figures 1E–G). Oil Red O staining (Figures 1H, I) confirmed these biochemical findings; cells treated with ox-LDL alone exhibited prominent, lipid-laden droplets, whereas catechin co-treatment markedly mitigated lipid accumulation. These results indicate that catechin counters ox-LDL-induced foam cell formation in MOVAS cells.

### 3.3 Catechin inhibits ferroptosis by reducing intracellular iron and ROS

**Figure 2:**
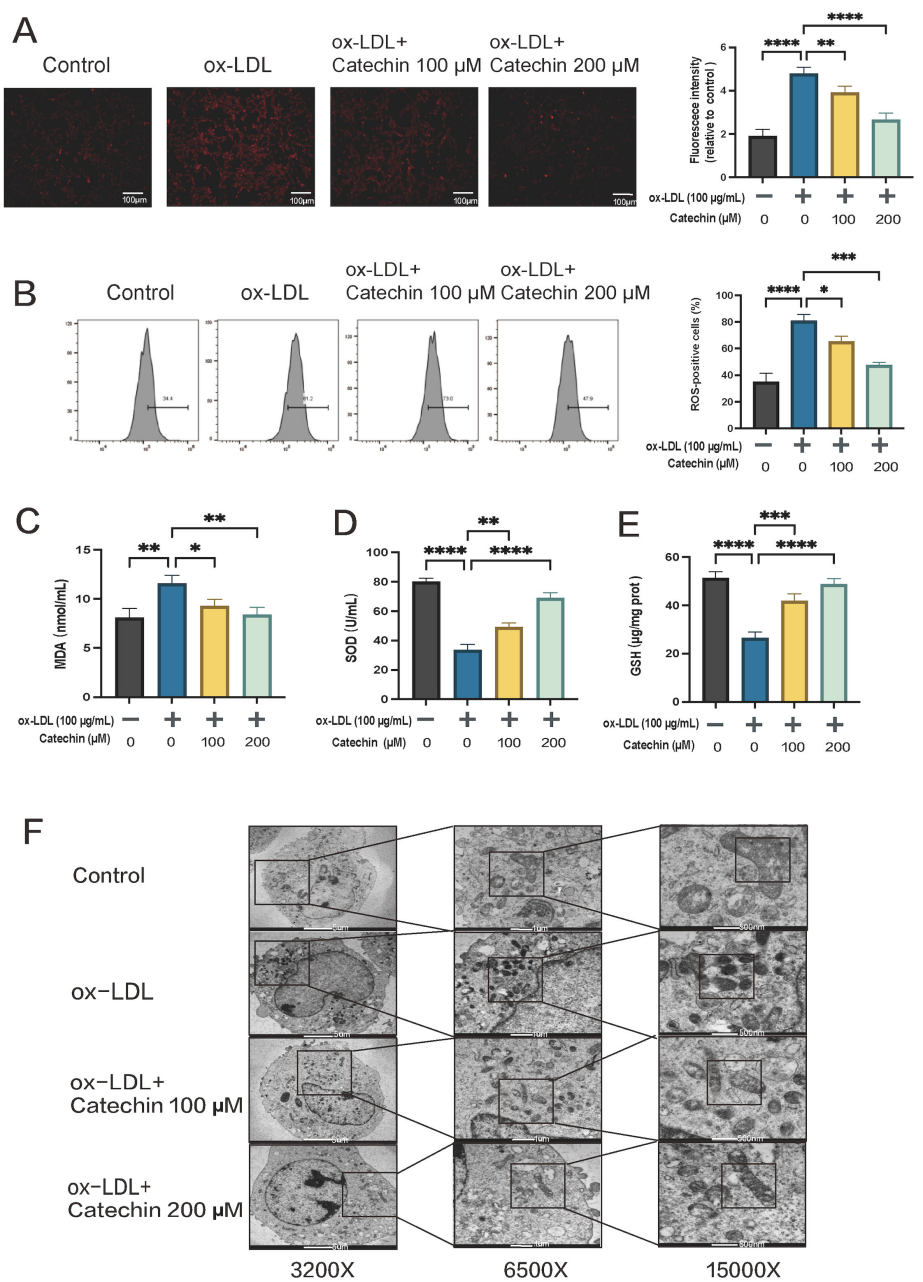
Catechin mitigates iron- and reactive oxygen species (ROS)-dependent oxidative stress in MOVAS cells. **(A)** FerroOrange staining reveals elevated Fe^2+^ in cells treated with 100 μg/mL ox-LDL, which is reduced by catechin in a dose-dependent manner. **(B)** DCFH-DA flow cytometry indicates that catechin treatment decreases oxLDL-induced ROS levels. **(C)** MDA levels, a marker of lipid peroxidation, are increased by oxLDL and lowered by catechin. **(D, E)** Catechin treatment restores SOD and GSH levels depleted by ox-LDL. **(F)** Transmission electron micrographs show mitochondrial condensation with collapsed cristae in the ox-LDL group. These changes are normalized by catechin. Data are mean ± SD (*n* = 5). *p < 0.05, **p < 0.01, ***p < 0.001, ****p < 0.0001.

To explore whether ferroptosis contributes to ox-LDL-induced VSMC dysfunction, we assessed intracellular Fe^2+^, ROS, and MDA. Excess ferrous iron can promote ROS generation via the Fenton reaction (30). Compared to controls, Fe^2+^ levels in ox-LDL-treated cells rose sharply (*p* < 0.01), but catechin (100 or 200 μM) significantly inhibited this increase (Figure 2A). Flow cytometric analysis with DCFH-DA revealed that ox-LDL elevated ROS production, which was reversed by catechin co-treatment (Figure 2B). Consistent with reduced ROS, MDA levels, an index of lipid peroxidation, were also decreased in the catechin groups (Figure 2C).

### 3.4 Catechin enhances antioxidant defenses and preserves mitochondrial integrity

We further examined whether catechin increases endogenous antioxidant capacity by measuring SOD and GSH. Ox-LDL exposure diminished SOD and GSH, but catechin restored their levels in a concentration-dependent manner (*p* < 0.05; Figures 2D–E). TEM images revealed that mitochondria in the ox-LDL group were shrunken with disrupted cristae, consistent with ferroptotic injury. By contrast, mitochondria in catechin-treated cells were largely intact (Figure 2F). This preservation of mitochondrial structure underscores catechin's protective effect against ferroptosis-associated damage.

### 3.5 Catechin restores GPX4, SLC7A11, and Nrf2 expression

**Figure 3:**
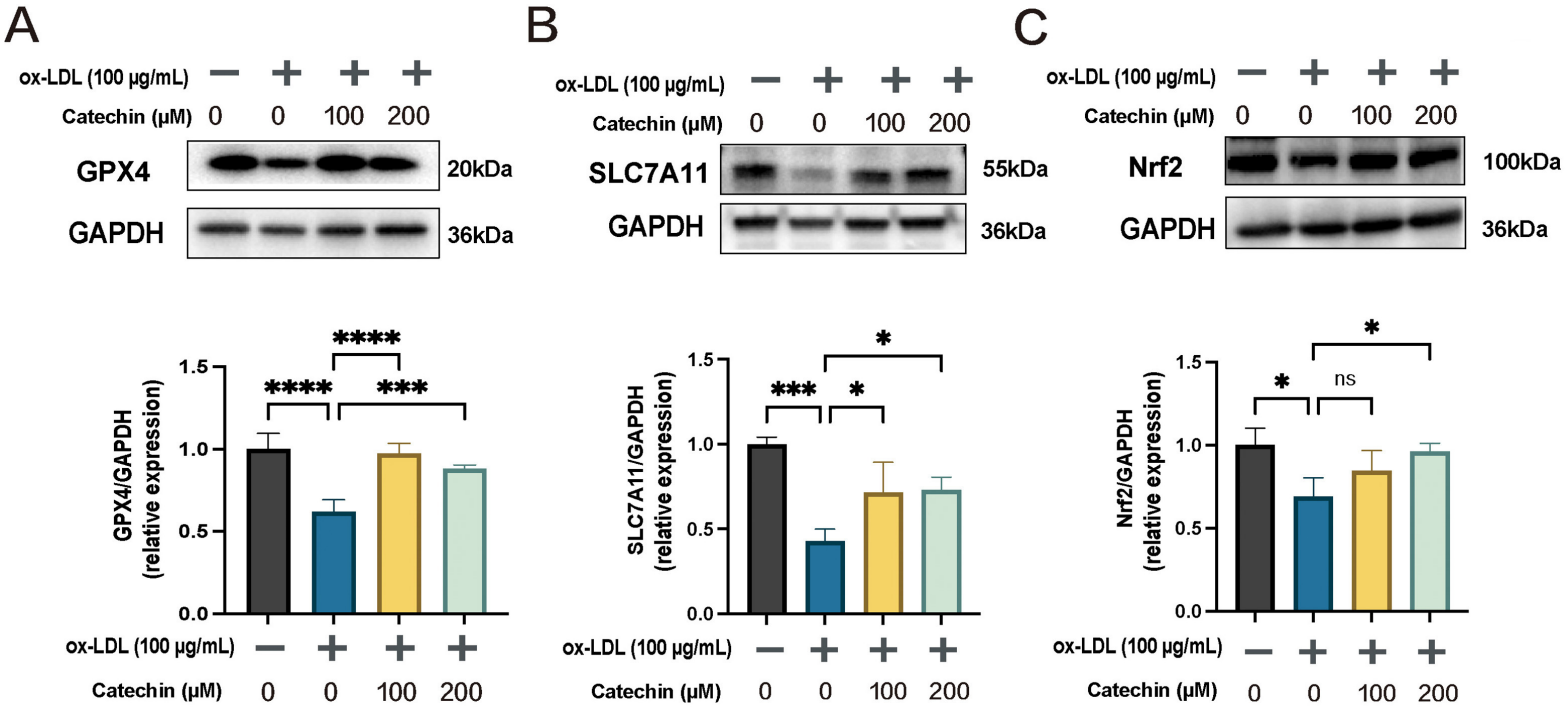
Catechin rescues ferroptosis-related protein expression in MOVAS. Western blot and quantification show that ox-LDL downregulates GPX4 **(A)**, SLC7A11 **(B)**, and Nrf2 **(C)**. Catechin substantially reverses this effect, with the effect on Nrf2 being most pronounced at 200 μM. Data are mean ± SD (*n* = 5). ns, not significant; *p < 0.05, ***p < 0.001, ****p < 0.0001.

To confirm catechin's anti-ferroptotic mechanism, we evaluated GPX4, SLC7A11, and Nrf2 by Western blot (Figure 3). Ox-LDL reduced GPX4 and SLC7A11 in MOVAS cells, whereas catechin significantly upregulated these proteins (*p* < 0.01). Notably, catechin at 200 μM also restored Nrf2, suggesting that higher doses more effectively engage the Nrf2 pathway. Collectively, these data provide mechanistic support that catechin abrogates ferroptosis by fortifying the Nrf2/SLC7A11/GPX4 axis.

### 3.6 Catechin alleviates atherosclerotic lesions in ApoE^−/−^ mice after partial ligation

**Figure 4:**
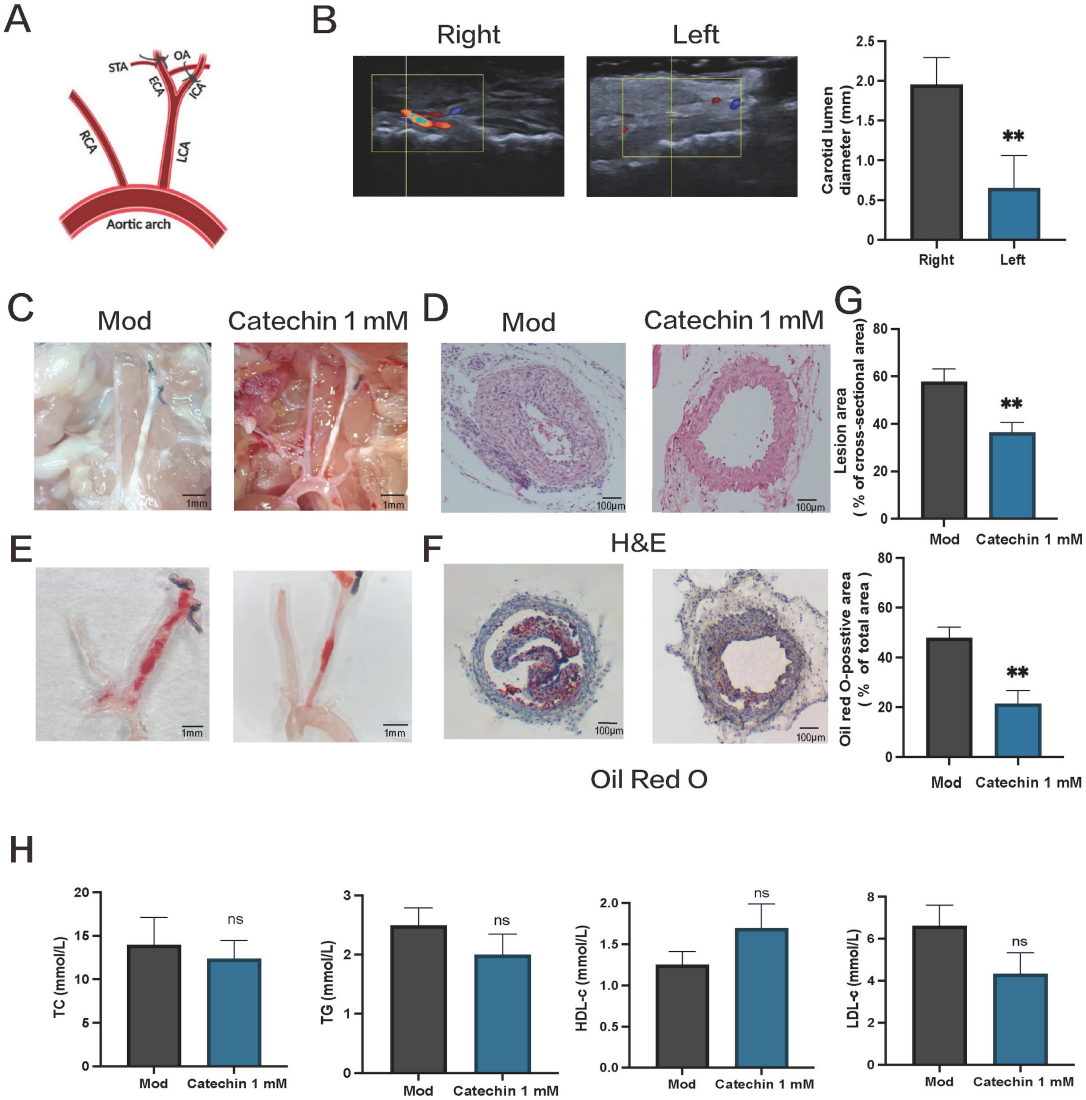
Catechin reduces atherosclerotic lesion formation in ApoE^−/−^ mice subjected to partial carotid ligation. **(A)** Schematic of partial ligation and local catechin delivery, as detailed in the Methods. **(B)** Ultrasound imaging reveals diminished blood flow and a reduced arterial lumen diameter in the ligated LCA. **(C, D)** Representative micrographs showing plaques in carotid arteries **(C)** and hematoxylin and eosin (H&E) staining of tissue sections **(D)** and Oil Red O staining **(E, F)** en face and of cross sections of ligated arteries demonstrate smaller plaques and reduced lipid accumulation in catechin-treated (CAT) mice compared to mock-treated (MOD) mice. **(G)** Shows quantification of lesion burden and lipid content in the ligated arteries. **(H)** Serum lipid profiles, including total cholesterol (TC), triglycerides (TG), high-density lipoprotein cholesterol (HDL-c), and low-density lipoprotein cholesterol (LDL-c), are unchanged. Data are mean ± SD (*n* = 5). ns, not significant; **p < 0.01.

We next tested catechin in ApoE^−/−^ mice subjected to partial LCA ligation and fed a high-fat diet for 2 weeks (Figure 4A). Ultrasound imaging confirmed reduced flow in the ligated LCA (Figure 4B). En face Oil Red O staining revealed substantially smaller plaques in catechin-treated mice, and cross-sectional H&E and Oil Red O staining indicated decreased lipid accumulation and lesion size (Figures 4C–G). Systemic lipid levels remained unchanged, presumably due to local catechin application (Figure 4H). These data mirror our *in vitro* findings, indicating that catechin stabilizes atherosclerotic plaques under disturbed flow conditions.

### 3.7 Catechin upregulates ferroptosis-related proteins in injured arterial tissue

**Figure 5:**
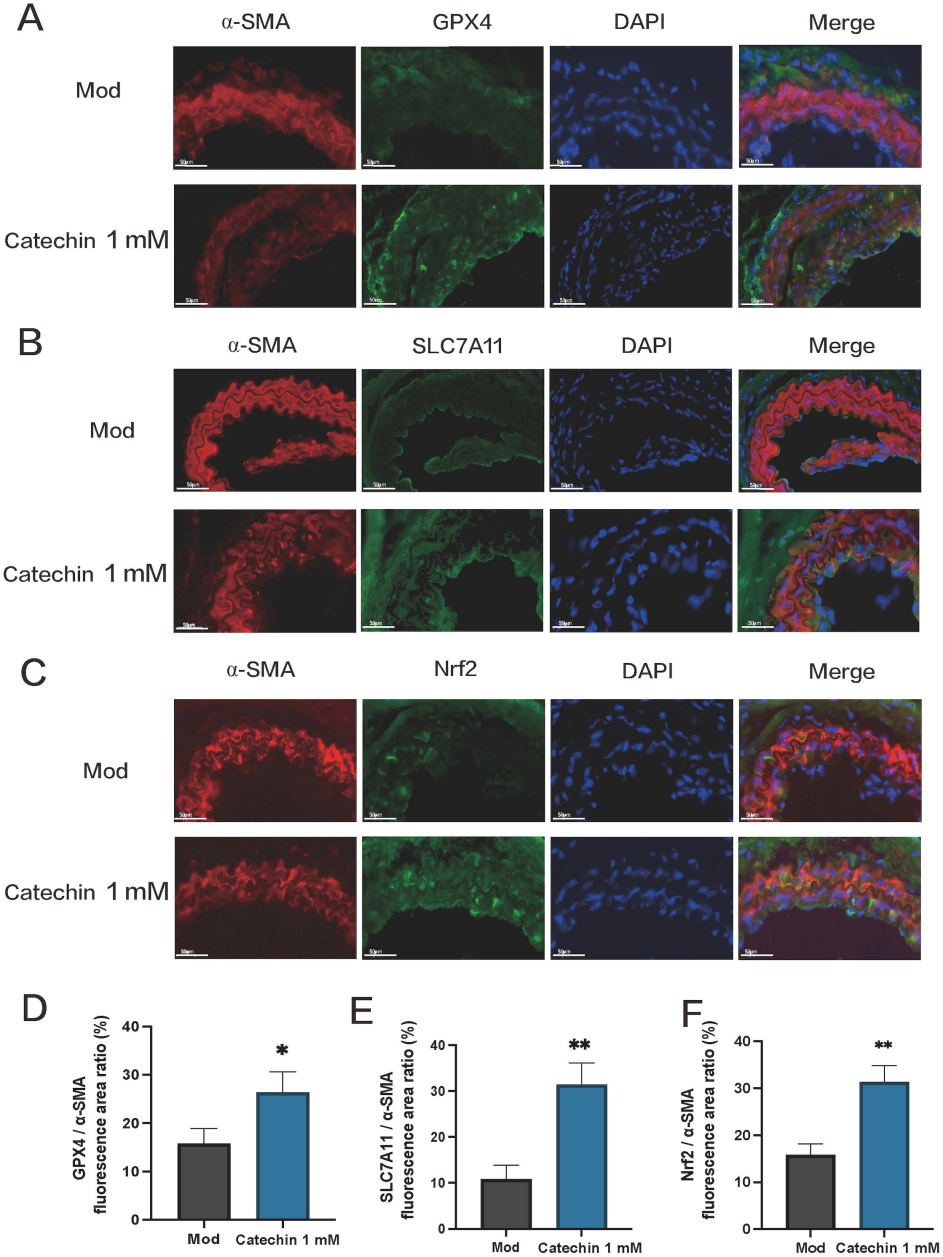
Catechin upregulates ferroptosis-related proteins in arteries. **(A–C)** Immunofluorescence of arterial sections shows alpha-smooth muscle actin (α-SMA, red) co-expression with GPX4 **(A)**, SLC7A11 **(B)**, or Nrf2 **(C)** (green) in both mock (Mod) and catechin-treated mice, with stronger immunoreactivities for these markers in catechin-treated arteries, consistent with reduced ferroptotic stress and more stable lesions. **(D–F)** Graphs depicting smooth muscle expression of GPX4, SLC7A11, and Nrf2, presented as the ratio of each marker's fluorescent signal to that of α-SMA. Data are mean ± SD (*n* = 5). *p < 0.05, **p < 0.01.

Immunofluorescence in arterial sections showed α-SMA (red) colocalized with GPX4, SLC7A11, and Nrf2 (green). Catechin-treated mice exhibited stronger GPX4/SLC7A11/Nrf2 signals than the mock treatment, suggesting reduced ferroptotic stress in the arterial wall (Figure 5). This *in vivo* upregulation aligns with the protective effects observed in MOVAS, providing translational evidence that catechin mitigates ferroptosis in atherosclerosis.

## 4 Discussion

Our findings demonstrate that catechin significantly inhibits ox-LDL-induced ferroptosis and foam cell formation in VSMCs, thereby reducing atherogenesis. Mechanistically, catechin decreased intracellular iron and ROS, increased GSH and SOD levels, and maintained mitochondrial architecture. Consistent with these outcomes, catechin also restored GPX4, SLC7A11, and Nrf2—the core ferroptosis defense components. These multifaceted antioxidant actions likely underlie catechin's plaque-reducing effects observed in ApoE^−/−^ mice after partial carotid ligation.

### 4.1 Ferroptosis and foam cell formation—exploring a causal link

An emerging body of work links ferroptosis to foam cell formation and atherosclerotic lesion progression (10–12, 31). However, the precise cause-effect relationship between ferroptosis and foam cell generation remains under investigation. Excess intracellular iron and ROS can oxidize lipids both within and outside cells, promoting the uptake of ox-LDL by smooth muscle cells or macrophages (32–34). In this manner, ferroptosis-associated oxidative stress can accelerate lipid loading and foam cell formation, suggesting that ferroptosis may potentiate or contribute to foam cell development. In addition, foam cell formation can lead to heightened oxidative stress within the plaque microenvironment—potentially creating a feedback loop that amplifies ferroptosis. Recent studies in macrophages have shown that iron accumulation and lipid peroxidation drive cell death, which in turn releases pro-inflammatory mediators that exacerbate plaque vulnerability (9–11). Although direct *in vivo* evidence mapping ferroptosis onset to foam cell emergence in VSMCs is limited, our findings in MOVAS cells indicate that ox-LDL-induced foam cell formation correlates with ferroptotic features (iron overload, mitochondrial shrinkage, and GPX4 depletion). Thus, ferroptosis and foam cell formation may operate in a vicious cycle: lipid loading triggers an oxidative environment that fosters ferroptosis, which further drives lipid accumulation and cell death. This interplay likely contributes to plaque expansion and destabilization.

### 4.2 Catechin's anti-ferroptotic and plaque-reducing effects

Our results demonstrate that catechin effectively attenuates ox-LDL-induced ferroptosis in VSMCs by lowering intracellular iron (Fe^2+^) and ROS, reducing lipid peroxidation, and upregulating core protective proteins (GPX4, SLC7A11, Nrf2). Importantly, catechin also inhibited foam cell formation, suggesting it disrupts the iron-ROS amplification loop that underpins ferroptosis. In ApoE^−/−^ mice, local delivery of catechin confirmed these protective effects at the arterial level, reducing lesion size and plaque lipid content. Hence, catechin may serve as an adjunct to conventional treatments, targeting both lipid overload and ferroptosis-mediated vascular injury (20).

### 4.3 Mechanistic considerations and future directions

Several mechanisms may explain how catechin inhibits ferroptosis and foam cell accumulation. First, catechin's direct ROS scavenging and mild iron-chelating properties can reduce the catalytic drive of the Fenton reaction (9, 35). Second, by boosting Nrf2 signaling, catechin upregulates key antioxidant genes (e.g., SLC7A11, GPX4), reinforcing GSH-dependent detoxification of lipid peroxides (16, 36). Third, broader anti-inflammatory actions may also stabilize the fibrous cap by preserving VSMC viability. Future studies should clarify the precise spatiotemporal relationship between ferroptosis onset and foam cell formation in VSMCs, potentially utilizing genetic or pharmacological modulators to dissect how ferroptotic pathways influence lipid uptake and plaque composition. Additionally, investigating potential synergies with lipid-lowering or anti-inflammatory agents reveals complementary therapeutic strategies for late-stage atherosclerosis.

## 5 Conclusion

Catechin effectively suppresses ox-LDL-induced ferroptosis in VSMCs by decreasing intracellular iron overload, ROS, and lipid peroxidation, while enhancing the Nrf2/SLC7A11/GPX4 ferroptosis defense system. Our data suggest that ferroptosis contributes to foam cell formation and atherosclerosis progression, although the exact causal sequence warrants further study. *In vivo*, local catechin delivery reduces plaque burden in ApoE^−/−^ mice. These findings underscore ferroptosis as a promising therapeutic target in atherosclerosis and identify catechin as a viable anti-ferroptotic, plaque-stabilizing agent.

## Data Availability

The original contributions presented in the study are included in the article/supplementary material, further inquiries can be directed to the corresponding author.
